# Supplementary material for: On the Oral Microbiome of Oral Potentially Malignant and Malignant Disorders: Dysbiosis, Loss of Diversity, and Pathogens Enrichment
Source: Int J Mol Sci. 2023 Feb 9;24(4):3466. doi: 10.3390/ijms24043466 (PMC9961214; doi:10.3390/ijms24043466)
Supplement: Supplementary file 1 [file ijms-24-03466-s001.zip › Supplementary Data S1.pdf]

Supplementary Data S1. Clinicopathological information of the OSCC patients with or without prior history of PVL included in the study.[a.]

Characteristics	Control		HL		PVL		OSCC		OSCC-PVL	
	N= 11	%	N = 9	%	N = 12	%	N= 10	%	N= 8	%
**Age at diagnosis**	67.91 ± 12.00		66.33 ± 8.12		66.83 ± 12.16		72.80 ± 9.05		80.25 ± 12.42	
**Gender**										
Male	5	45.5	4	44.4	3	25.0	6	60.0	6	75.0
Female	6	54.5	5	55.6	9	75.0	4	40.0	2	25.0
**Tobacco smoking**										
Yes	2	18.2	4	44.4	5	41.7	4	40.0	1	12.5
No	9	81.8	5	55.6	7	58.3	6	60.0	7	87.5
**Alcoholism**										
Yes	0	0.0	2	22.2	1	8.3	1	10.0	0	0.0
No	11	100.0	7	77.8	11	91.7	9	90.0	8	100.0
**Sampling location**										
Gingiva	8	72.7	1	11.1	3	30.0	3	30.0	5	62.5
Palate	0	0.0	1	11.1	1	10.0	1	10.0	2	25.0
Floor of mouth	0	0.0	2	22.2	1	10.0	1	10.0	0	0.0
Tongue	1	9.1	3	33.3	4	40.0	4	40.0	0	0.0
Buccal mucosa	2	18.2	1	11.1	0	0.0	0	0.0	1	12.5
Lips	0	0.0	1	11.1	1	10.0	1	10.0	0	0.0
**Dysplasia of the non-neoplastic lesions**										
Absence			2	22.2	3	25.0				
Mild			5	55.5	7	58.4				
Moderate			2	22.2	1	8.3				
Severe			0	0.0	1	8.3				
**Clinical form of the OSCC**										
Erythroplastic							0	0.0	2	25.0
Ulcerated							9	90.0	1	12.5
Exophytic							0	0.0	3	37.5
Mixed							1	10.0	2	25.0
**Differentiation grade**										
G0							0	0.0	2	25.0
G1							5	50.0	4	50.0
G2							4	40.0	1	12.5
G3							1	10.0	1	12.5
**Cancer infiltratation**										
Bone							3	30.0	3	37.5
Perineural							7	70.0	2	25.0
Lymphovascular							2	20.0	2	25.0
No							0	0.0	1	10.0
**Lymph node metastasis**										
Yes							3	30.0	1	12.5
No							7	70.0	7	87.5
**Distant metastasis**										
Yes							1	10.0	0	0.0
No							9	90.0	8	100.0
**TNM staging**										
I							2	20.0	1	12.5
II							2	20.0	3	37.5
III							0	0.0	1	12.5
IV							6	60.0	3	37.5
**Second primary tumors**										
Yes							2	20.0	4	50.0
No							8	80.0	4	50.0

[a]No significant differences were found in the clinicopathological characteristics of patients.
