# Supplementary material for: On the Oral Microbiome of Oral Potentially Malignant and Malignant Disorders: Dysbiosis, Loss of Diversity, and Pathogens Enrichment
Source: Int J Mol Sci. 2023 Feb 9;24(4):3466. doi: 10.3390/ijms24043466 (PMC9961214; doi:10.3390/ijms24043466)
Supplement: Supplementary file 1 [file ijms-24-03466-s001.zip › Supplementary Data S2.pdf]

Supplementary Data S2. Alpha-diversity indexes for oral samples collected from healthy controls and HL, PVL, OSCC and PVL-OSCC patients. Asterisk indicates $p < 0.05$.

**Variable**	**Control (n=11)**<br>Mean (SD)	**OSCC (n=10)**<br>Mean (SD)	**HL (n=9)**<br>Mean (SD)	**PVL (n=12)**<br>Mean (SD)	**PVL-OSCC (n=8)**<br>Mean (SD)	***P*-value**
Chao1	637.94 (306.85)	284.18 (166.95)	565.53 (295.83)	652.73 (476.71)	320.56 (244.04)	0.031*
Shannon	4.6 (1.04)	3.53 (0.74)	4.23 (0.95)	4.39 (1.06)	3.68 (0.87)	0.064
Simpson	0.07 (0.04)	0.06 (0.02)	0.05 (0.03)	0.06 (0.03)	0.07 (0.03)	0.748
Inverse Simpson	50.32 (45.72)	18.55 (17.66)	31 (30.43)	34.3 (29.64)	22.92 (20.11)	0.189

HL, homogeneous leukoplakia; PVL, proliferative verrucous leukoplakia, OSCC, oral squamous cell carcinoma; PVL-OSCC, oral squamous cell carcinoma preceded by proliferative verrucous leukoplakia
