# Supplementary material for: On the Oral Microbiome of Oral Potentially Malignant and Malignant Disorders: Dysbiosis, Loss of Diversity, and Pathogens Enrichment
Source: Int J Mol Sci. 2023 Feb 9;24(4):3466. doi: 10.3390/ijms24043466 (PMC9961214; doi:10.3390/ijms24043466)
Supplement: Supplementary file 1 [file ijms-24-03466-s001.zip › Supplementary Data S3.html]

Javascript must be enabled to view this page.

magnitude
magnitudeUnassigned

Control
HL
OSCC
PVL
PVL-OSCC

301467321701576672490749337270

19278168088265213863460
298526319402575959487139336593

12944835

1099435

1099435

305

305

10

10

10

4
784435

772

1235

1708

1708

1708

1708

1708

25

25

25

25

143

14

14

14

3

3

3

3

43

43

43

43

43

9305831711

9305831711

9305831211

9305831211

9305831211

5

5

5

1510066782452371132950

1510066782452371132950

1510066782452371132950

183
1510066782452371132950

11753924972571513850
1487266775782371132950

17343112752

176337081

19

170364631178

21435

282016417314112766

3703462199137262595

32941311622082435

74

267

95119111

1252

1073

45667

45667

55865831948118113549769746
3

4887032
3809970773385148156322296

76374695660192

69374669653192

50248

50248

33366547613184

16

36872

7267

267

7

3128165784157576876911929
61115630349730

2941361837144476119511233

2939661825144476119511233
2910949243139125490910080

1488289684758392

1251205391

50

7742932121110163

204213207

1712

112

112

112

1113

1113

51448272429

51448253429

19

52232467406873654

52232467406873654

21063701623

21063701623
21063621623

5

3

18703316

18703316

33

187016

16

1865

5

12

12

12

8971211607320348071

8971211607320348071

8971211607320348071
211

93063793876

16044102

354105100

5431666673092

340044735814100582088

319643814818100582088

319643814818100582088

20492996

20492996

494584

494584

494584
494582

2

33597

33597

1997

145

379

379

379

379

379

81493902262283233730048
35

36162283198

36162283198
7

29162283198

32682503156051130017647

1100271391661815

1100271391661815

1100271391661815

21682476154661113415832
29262661954

1155

85109

85109

14

210

4955

5432202144522159
5342201059522159

91085

493674630486811826
394634630481311686

3

99455137

8

8

1264

1264

169128532281783108

26

193619207

193619207

2946935753551172

28883316272466233
28883316272454233

12

280

280

9

188387284645739

188387284645739

188387284645739
71317260547639

117702498100

400993896161858311291
455236

3255339821852
2238269756049957723

642311
207416913750479

207416913108168

1318642289

3935822022681

3935822022681

7810014701057422
781001470636422

421

2

939281178185193031

1619

3133490
3168490

35

63628115027761020
2892811502776558

347462

465231
468041368

40

683

33

726454

13965

25497

311979456

4887

115281216
832388275517501

397

3632
13246292428

9646292048

6

71401744
561441744

494

18489134503
4

22631

9911153

63

72134

27
312415957

52414130

2618

30381592220

263

4081592220

11

431535611398171

431535611398171

4
431535611398171

431535611394171

4

4

4

4

1351146

1351146

1351146

7510772

48362
209

152

1027

3

25
201041

6

6

5

57

935

7

7

7

92358519164392159717402

92358519164392159717402

5885834113377130671688

6

6

11512744

11512744

69110550957

69110550957

318679140181642
36082925443858962

209

162

4222134041833758

2035687564957748653
20936932128208656669

35899

58226325916

1641466228034149
33501783062853015714

140

27

492976212911698

2970135163844369867
1438473592049339

41108111873

4155

5

561647

34582

1000359637778793

3881223380

1344192

58707804771114675560236752
38825899521185

55546779211105645157135913

28641153187193

28641153187193

2

132345349165
28441153187193

152710013828

776

776

776

28

28

28

93841706051042643214493

349445
15

7

445

445

12

12

9

541226442014

541226442014

87921683450958636814474
119228277

10461452292253

23191970

3471138683374821882477
69231662748145590510403

169161931350493

87534667

6141365159944

258224091336927017389

6561725021731464
5971725021731464

59

1584
481584

48

1031

10

31

7

7

7

206

147

147

59

59

4573160816472214068917818

15
4573160816472214068917818

677568104243
59227

61856810416

4397259995389313439313250
4377659529373213423212527

2227

90885230

698189

106444161304

1058252827961874095
1082252829061874325

24

11230

548

548
4

5

5

44

14041007142601545

489788368

489788368

92428342601177

1811

66

37259
13259

24

4931177

44246

44246

101931864
27

181

181

239

5

234

48

48

94431444
8871444

3

57

2024511115
277322988043510654

269622237563240600

269622237563240600

269622237563240600

5
11

6

6

282520

39232713219

39232713219

12

12

9

56

342313219

2

2

716

716

716

35368537274332

35368537274332

35368537274332

35368537274332

4

4

91294152

91294152

3445857859128

2246432187810032775

1241401250243347

1241401250243347

52
930262244192181

1021766281181

137642680

6392215631

311139651166

311139651166

29721

14118651166

30

30

30

30

9753116287602428

10149

10149

10149

10149

145

145

145

145

9753116047602274

9753116047602274

9753116047602274

9753116047602274

73

73

73

73

73

73

62831431631458749630242487

62831431631458749630242487

13723894
5407939285615428855531063

161

161

2338

1224
2338

1114

23563221791181113

23563221791181113

191721376

191721376
19526

6

121364

183354975731
1418129794397384967221049

1010525861261084317012706
2362169757934441723

76536

4323215

89482

87

99567887816157682705

74726

86716180565186

8327

1851422413

89732541

606

55043451944182

19110273185

11924186

667353904

4210

35150333428

15215

596303789352124

2780130592286

199366928

10467

1833779256

28124115

51110467106384

20912335445090565

789108942982411967

3054627

24134828981013

66171341857

3691088

3805105

29638401245241646392
222939261305757258312

1602865511428986

33154133934

1472420

1472420

898152092

4519
898152092

5

9

839152073

758372336112576410

758372336112576410
38726672800584328

371156811199282

25933837017404304669353

25933837017404304669353
3871442112497170112496

20336749

43715451912

68544406

2258

1957114599

64

27938532211322213

2

19783839210846818

2833443745944

4
225843728944

58

129843714625

3813919

5026170

8

26421034457

26421034457

26421034457

2577432133401

2577432063401

2577432063401
2

2575432063401

7

7

6

6

6

5

5

5

616938358411477478023

534926217206265824649

14751144430479665
534926217206265824649

129267971975405

89420

1394124396582723246

536

4003161825126764

52118002515822792569

82012141205211653374

80812141205211653374

8

4

302174589077341845

262273037657213775

110836541

110836541

35925

235
110530616

18

5

12

11053616

47347818

47347818

824

824

3
3910318

391018

75

75

2107030284

4

2106630284

2106630284

97568603216497461

97568603156497461
3

23

97568602926494461
88763102926494461

88550

6

6

28237315569292

28237315569292

213
65

148

1333731556979
3914715137

40

18

1619240139

30

20

343110

149

39915514212870

39915514212870

39915513812870

20219
191

11

19

19715513810970
17215513810363

25

6

7

4

4

712837852319504193315101640

712837852319504193315101640

712837852319504193315101640

40382614811427666828686095

35738324931201725683678508
40382614811427666828686095

46442898822594114507587

3090117042522752502915545
13

66108
108

66

257

3088817042519522502915437
2138515587315022284512292

1542712

4871873507359

644

380254622391208

172

133813

3952590676738

27529891372

72385357606357655

52268561221385218445895

52268561221385218445895

52268561221385218445895

8

8

8

52268561221385218445887

52268561221385218445887
20341831700

100

32301252811223

920234

4381622667267396446

11272117662067115037

41396382

1771199702428862470

217317216092566448
294122997133610677

28626549332100

28626549332100

28626549332100

28626549332100

28626549332100

48231355712129
6025787

42228848625129

42228848625129

42228848625129

42228848625129
