# Supplementary figures and images for: On the Oral Microbiome of Oral Potentially Malignant and Malignant Disorders: Dysbiosis, Loss of Diversity, and Pathogens Enrichment
Source: Int J Mol Sci. 2023 Feb 9;24(4):3466. doi: 10.3390/ijms24043466 (PMC9961214; doi:10.3390/ijms24043466)

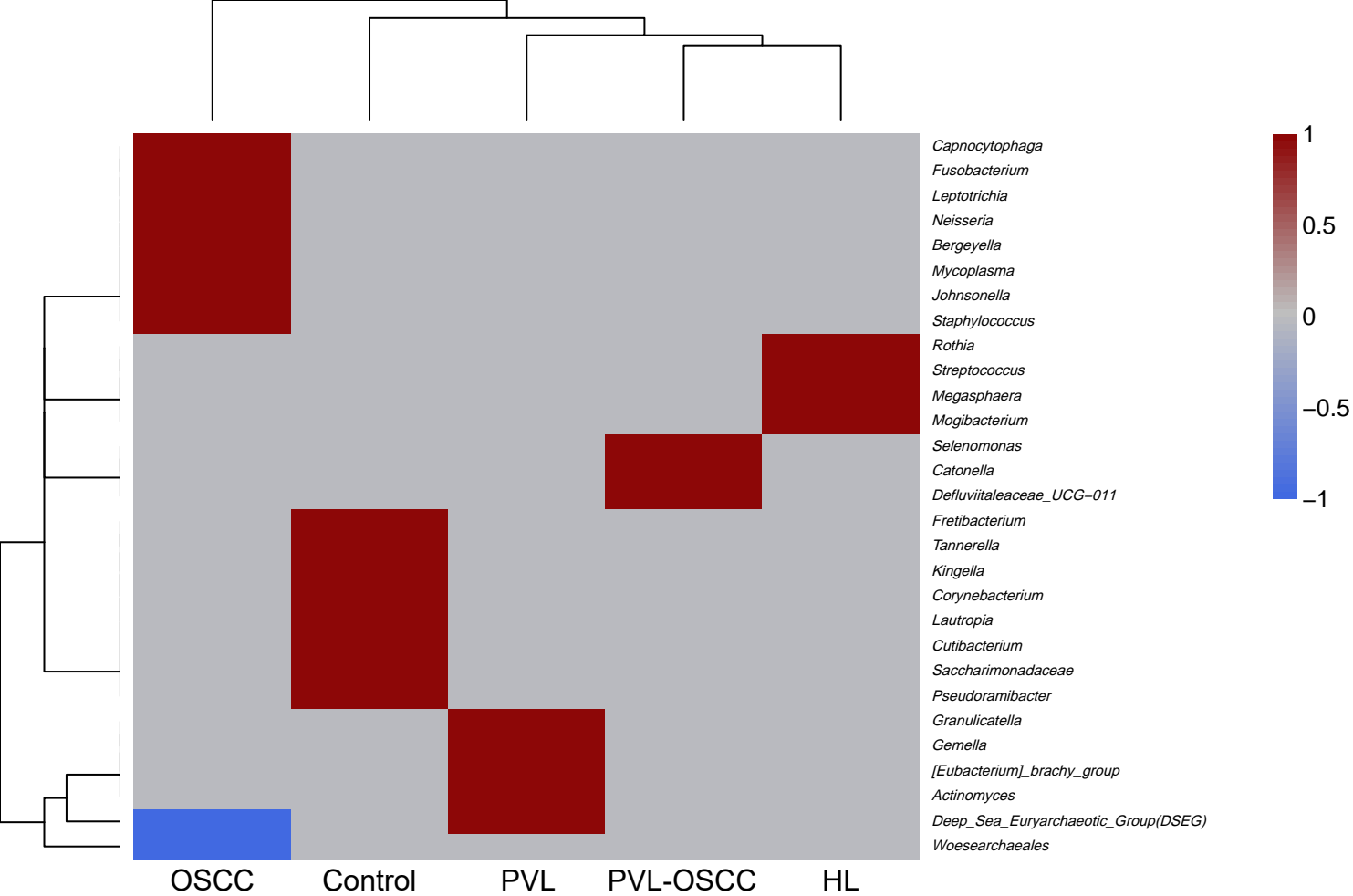

Supplement: Supplementary file 1 [file ijms-24-03466-s001.zip › Supplementary Data S4.pdf]

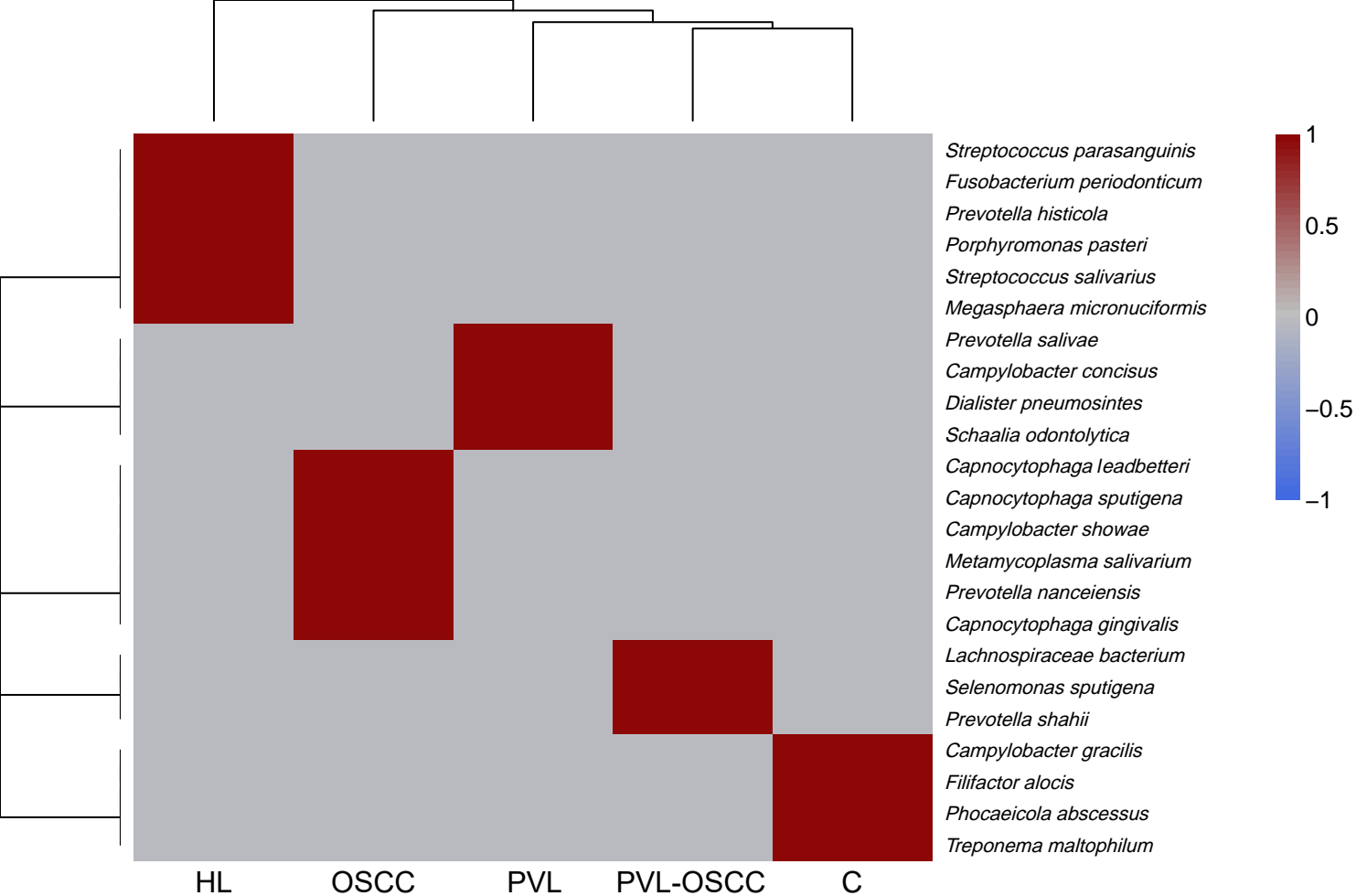

Supplement: Supplementary file 1 [file ijms-24-03466-s001.zip › Supplementary Data S5.pdf]
